# Low Concentration of Exogenous Carbon Monoxide Modulates Radiation-Induced Bystander Effect in Mammalian Cell Cluster Model

**DOI:** 10.3390/ijms17122051

**Published:** 2016-12-08

**Authors:** Wenqing Wu, Lili Nie, K. N. Yu, Lijun Wu, Peizhong Kong, Lingzhi Bao, Guodong Chen, Haoran Yang, Wei Han

**Affiliations:** 1 Center of Medical Physics and Technology, Hefei Institutes of Physical Science, Chinese Academy of Sciences, Hefei 230031, China; 18256986803@163.com (W.W.); niell@cmpt.ac.cn (L.N.); peter.yu@cityu.edu.hk (K.N.Y.); ljw@ipp.ac.cn (L.W.); gckongpz@163.com (P.K.); lingzhibao@hfcas.ac.cn (L.B.); c3024034@163.com (G.C.); yhr2853@163.com (H.Y.); 2 Department of Physics and Materials Science, City University of Hong Kong, TatChee Avenue, Kowloon Tong, Hong Kong, China; 3 Institute of Technical Biology & Agricultural Engineering, Hefei Institutes of Physical Science, Chinese Academy of Sciences, Hefei 230031, China; 4 Collaborative Innovation Center of Radiation Medicine of Jiangsu Higher Education Institutions and School for Radiological and Interdisciplinary Sciences (RAD-X), Soochow University, Suzhou 215000, China

**Keywords:** low centration of carbon monoxide, radiation-induced bystander effect, cell cluster model, inducible nitric oxide synthase, cyclooxygenase-2

## Abstract

During radiotherapy procedures, radiation-induced bystander effect (RIBE) can potentially lead to genetic hazards to normal tissues surrounding the targeted regions. Previous studies showed that RIBE intensities in cell cluster models were much higher than those in monolayer cultured cell models. On the other hand, low-concentration carbon monoxide (CO) was previously shown to exert biological functions via binding to the heme domain of proteins and then modulating various signaling pathways. In relation, our previous studies showed that exogenous CO generated by the CO releasing molecule, tricarbonyldichlororuthenium (CORM-2), at a relatively low concentration (20 µM), effectively attenuated the formation of RIBE-induced DNA double-strand breaks (DSB) and micronucleus (MN). In the present work, we further investigated the capability of a low concentration of exogenous CO (CORM-2) of attenuating or inhibiting RIBE in a mixed-cell cluster model. Our results showed that CO (CORM-2) with a low concentration of 30 µM could effectively suppress RIBE-induced DSB (p53 binding protein 1, p53BP1), MN formation and cell proliferation in bystander cells but not irradiated cells via modulating the inducible nitric oxide synthase (iNOS) andcyclooxygenase-2 (COX-2). The results can help mitigate RIBE-induced hazards during radiotherapy procedures.

## 1. Introduction

Radiation-induced bystander effect (RIBE) creates potential genetic hazards for normal tissues surrounding the targeted regions during radiotherapy procedures, and has been considered to have a close relationship with radiation-induced secondary cancers beyond the irradiated areas after radiotherapy [1,2]. RIBE refers to the phenomenon where irradiated cells release some signaling molecule(s) to act on neighboring non-irradiated cells through diffusion in the medium or cellular gap-junction communication, and has been extensively studied in the past decades since its discovery in 1992 [1]. RIBE causes cytotoxicity or genotoxicity in the non-irradiated cells that are similar to those observed in the irradiated cells [3]. In particular, significant increases in gene mutations [4,5], DNA damage [6,7], cell proliferation [8], chromosomal damage [9], neoplastic transformation [10] and even tumor formation [11] were observed in bystander cells or tissues in in vitro and in vivo studies. Further studies showed that RIBE intensity, for example in reference to the frequency of gene mutation, was much higher in cell cluster models than in monolayer cultured cell models [5,12]. RIBE also has been shown to induce positive effects. For example, RIBE reduced neoplastic transformation [13], and overexpressed protective proteins in bystander fish [14] and in rats [15]. RIBE-induced adaptive responses in the bystander cells have also been found [16,17,18]. The contradictory biological effects induced by RIBE highlight the complicated mechanisms underlying RIBE and further research is required for clarification.

Low-concentrations of CO can affect biological functions through binding to the heme domain of proteins and then modulating various signaling pathways [19]. The protective effect of exogenous CO against genotoxicity of RIBE in monolayer cells was first reported in our previous studies [20,21,22]. The relatively low concentration (14 µM) exogenous CO effectively attenuated the formation of RIBE-induced DNA double-strand breaks (DSBs) and chromosome breaks (surrogated by the micronucleus, MN) through reducing the amount of excessive O_2_^−^ or nitric oxide (NO) in the non-irradiated bystander cells, but there were no significant changes in the irradiated cells themselves [20,21,22].

**Figure 1:**
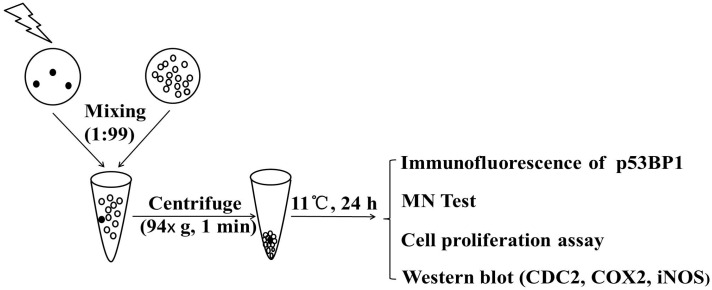
Schematic representation of multicellular cluster. ●: fluorescence labelled cells; ○: non-fluorescence labelled cells. CDC2: cell division cycle 2; COX-2: cyclooxygenase-2; iNOS: nitric oxide synthase; MN: micronucleus; p53BP1: p53 binding protein 1.

In the present paper, we further investigated the attenuation or inhibition of RIBE-induced cell proliferation and genetic damage in a model of mixed cell cluster (shown in Figure 1), which was developed by Eric Hall’s group for RIBE study [5] using low concentrations of exogenous CO. With sustained proliferation, gene mutations and chromosomal instability are considered to be risks for tumorigenesis [23]; our results will provide important information to protect normal tissues from RIBE hazards during radiotherapy procedures.

## 2. Results

### 2.1. CO (CORM-2) Decreased DSB Formation in the Bystander but Not Irradiated Cell Population

**Figure 2:**
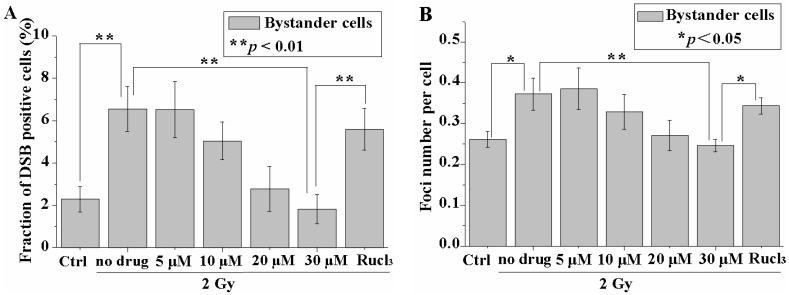
CO decreases double-strand breaks (DSBs) in bystander cells. Fraction of p53BP1-positive cells (**A**); and foci number per cell (**B**) in the bystander cells with or without CO (tricarbonyldichlororuthenium, CORM-2) treatment. Data are pooled from at least three independent repeats and the results are presented as mean ± SD. Significances in the differences between the samples are determined and differences with *p* < 0.05 are considered statistically significant. (* *p* < 0.05; ** *p* < 0.01).

The amount of p53 binding protein 1 (p53BP1) was measured at 5 h after the mixed cells were resuspended and plated on the cover glasses. As shown in Figure 2A, a significant increase in the fraction of p53BP1 positive cells and the p53BP1 foci number per cell compared with control were observed in the bystander cell population, the latter having been incubated with cells irradiated with a dose of 2 Gy. To confirm whether RIBE signal(s) had been completely transduced in the cell-cluster model, we centrifuged only the irradiated cells to form a cell cluster for 24 h at 11 °C, and then resuspended and mixed the irradiated cells with the non-irradiated bystander cells. Under such conditions, the fraction of p53BP1 positive cells and the number of foci per cell in the bystander cell population were 1.57% ± 0.68% and 0.197 ± 0.03, respectively, and these values were similar to those for the control bystander cells (i.e., 1.57% ± 0.49% and 0.215 ± 0.0126, respectively). These results indicated that the RIBE signal(s) had been completely transduced during incubation of the mixed multicellular cluster and no more RIBE took place after the cell cluster was re-suspended. 

The fraction of p53BP1 positive cells was found to decrease with the increasing concentration of CO (tricarbonyldichlororuthenium, CORM-2). In particular, CORM-2 with a concentration of 30 µM reduced the fraction of p53BP1 positive cells back to the control level. The number of p53BP1 foci also decreased in a concentration-dependent manner with the treatment of CO (CORM-2; Figure 2B). In addition, treatment with ruthenium trichloride (RuCl_3_, 30 µM) did not significantly decrease the fraction of p53BP1 positive cells in the bystander cell population (0 or 2 Gy) when compared to those cells without treatment with the chemical. 

No significant changes in the fraction of p53BP1 positive cells and in the number of foci per cell were observed in the cells treated only with 30 µM CO (CORM-2) (2.1% ± 0.5%; 0.27 ± 0.027) or in the cells treated with 30 μM RuCl_3_ (2.1% ± 0.7%; 0.27 ± 0.087) when compared to the control cells (1.9% ± 0.4%; 0.30 ± 0.09).

### 2.2. CO (CORM-2) Decreased MN Formation in the Bystander Cell Population

**Figure 3:**
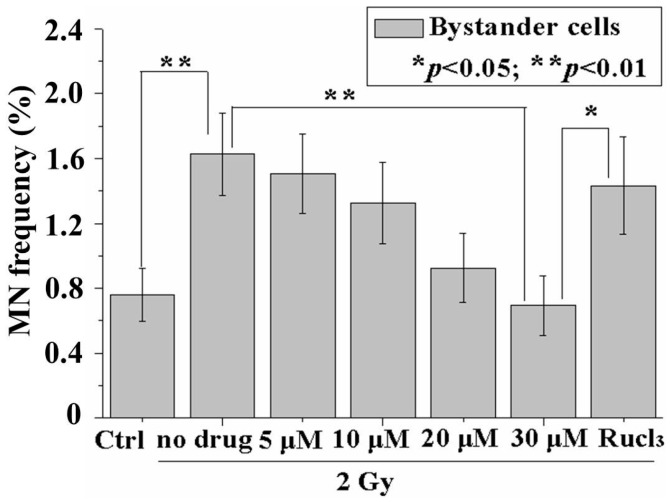
CO decreases micronucleus (MN) in the bystander cells. Data are pooled from at least three independent repeats and the results are presented as mean ± SD. Significances in the differences between the samples are determined and differences with *p* < 0.05 are considered statistically significant. (* *p* < 0.05; ** *p* < 0.01).

After 24 h incubation in 11 °C, the cell cluster was resuspended and the mixed cells were plated onto Petri dishes for MN assay. The results from MN assay showed similar trends with those from immunofluorescence of p53BP1. RIBE induced a significant increase in the MN frequency. The MN frequency was reduced to the background level (0.694% ± 0.185%) with 30 µM CORM-2 (Figure 3). On the other hand, no significant changes in the MN frequency were observed in the cells treated only with 30 µM CO (CORM-2) (0.5% ± 0.129%) or in the cells treated with 30 µM RuCl_3_ (0.549% ± 0.075%) when compared to the control cells without these treatments (0.59% ± 0.129%).

### 2.3. CO (CORM-2) Did Not Affect p53BP1 Formation and MN Frequency in Irradiated Cells

The effects of CO (CORM-2) on p53BP1 formation and MN frequency induced by direct radiation were also determined. No significant changes were observed in the fraction of p53BP1 positive cells, number of foci number per cell and MN frequency after treatment with 30 µM CO (CORM-2) when compared to those cells without chemical treatment (Figure S1).

In all subsequent experiments involving CORM-2, the concentration of 30 µM was used.

### 2.4. CO (CORM-2) Inhibited RIBE-Induced Cell Proliferation but Did Not Affect Irradiated Cells

**Figure 4:**
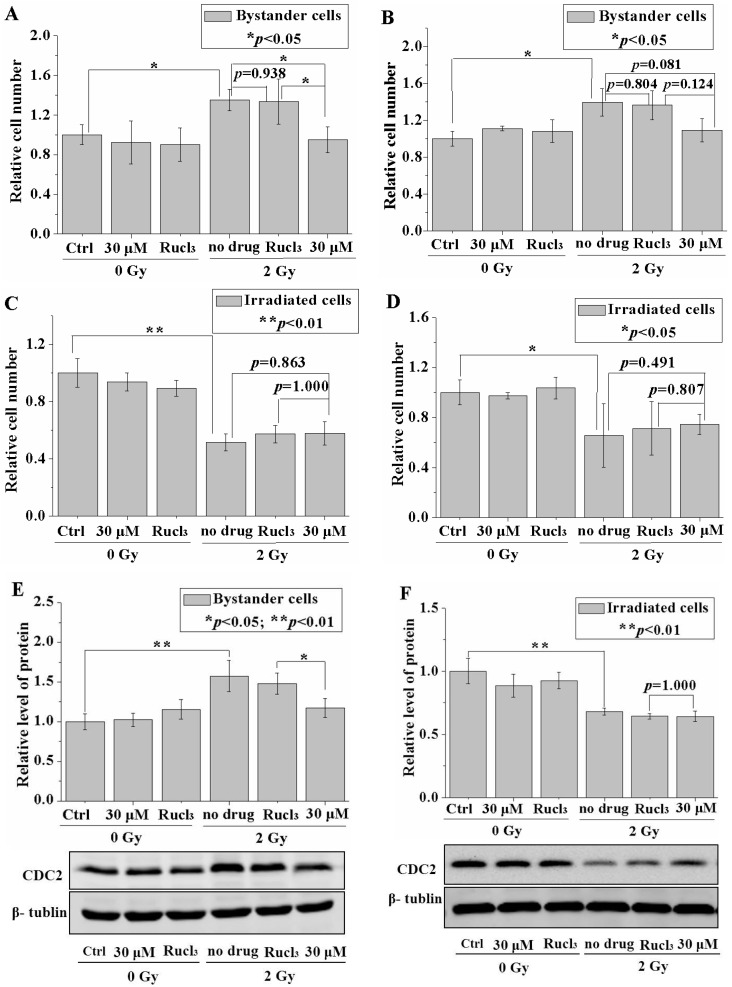
CO decreases proliferation of bystander cells. Relative cell number of bystander cells at 24 h (**A**) and 48 h (**B**) with or without treatment with 30 µM CO (CORM-2); relative cell number of irradiated cells at 24 h (**C**) and 48 h (**D**) with or without treatment with 30 µM CO (CORM-2) treatment; relative level of CDC2 protein expression in bystander (**E**) and irradiated (**F**) cells with or without 30 µM CO (CORM-2) treatment. Data are pooled from at least three independent repeats and the results are presented as mean ± SD. Significances in the differences between the samples are determined and differences with *p* < 0.05 are considered statistically significant. (* *p* < 0.05; ** *p* < 0.01).

Previous studies have reported RIBE-induced cell proliferation [8]. In the present experiment, the mixed cells in the cluster were re-suspended and plated onto 35 mm Petri dishes (3 × 10^5^ cells per dish). The cell number was measured at 24 or 48 h after cell plating. Figure 4A,B shows that the relative cell numbers (1.35- or 1.40-fold of the respective controls) were increased at 24 h (Figure 4A) or 48 h (Figure 4B). The results also showed that direct irradiation had inhibited the cell proliferation (0.51- or 0.66-fold of the respective controls) at 24 h (Figure 4C) or 48 h (Figure 4D) after cell plating. These results indicated that the number of mixed cells was increased due to RIBE-induced proliferation. Upon treatment with 30 μM CORM-2, the increased cell numbers at 24 or 48 h (Figure 4A,B) were reduced back to control levels (0.95- or 1.09-fold of the respective controls, respectively). Treatment with RuCl_3_ only but not CORM-2 did not significantly affect the proliferation of bystander cells. No distinct effects from treatment with 30 μM CO (CORM-2) were observed on the growth of cells irradiated with a dose of 2 Gy at 24 or 48 h (Figure 4C,D).

Gerashchenko et al*.* proved that a small fraction of irradiated cells (i.e., 5%) was good for a proteomic study of RIBE because the non-irradiated bystander cells were “contaminated” with only a very small number of irradiated cells [23]. Therefore, 1% irradiated cells in our mixed cell cluster would enable us to perform the western blot assay for the effect of CO on the protein expression in the bystander cells. The expression level of cell division cycle 2 (CDC2) cyclin-dependent protein kinase was also determined at 24 h after plating of the cells resuspended from the cluster. CDC2 was previously found to be highly expressed in proliferating bystander cells treated with the conditioned medium, which was harvested from the cells irradiated with low-dose α particles [8]. In the present experiments, a distinct increase (1.58 ± 0.2-fold of the non-irradiated control) in the CDC2 protein was detected in the mixed cells (ratio between the number of cells irradiated with a dose of 2 Gy and bystander cells = 1:99) (Figure 4E), but the expression of CDC2 protein decreased significantly in the cells irradiated with a dose of 2 Gy (Figure 4E). Upon treatment with 30 µM CO (CORM-2), the increased CDC2 expression induced by RIBE was significantly reduced to 1.17 ± 0.12-fold of the control. 

The CDC2 protein level was significantly decreased (to 0.68-fold of the control) in the irradiated cells, and treatment with 30 µM CO (CORM-2) did not significantly affect the CDC2 levels (Figure 4F).

### 2.5. Inhibition of iNOS or COX-2 Decreased RIBE-Induced p53BP1 Formation and MN Frequency

**Figure 5:**
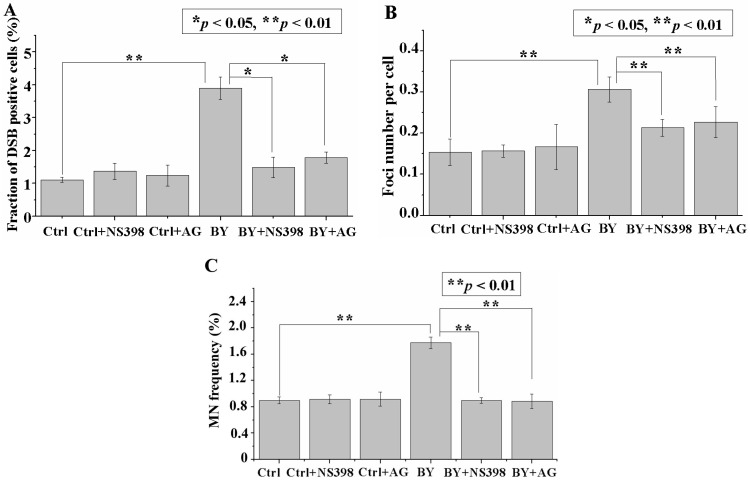
*N*-[2-(cyclohexyloxy)-4-nitrophenyl]-methanesulfonamide (NS 398) or aminoguanidine (AG) inhibits radiation-induced bystander effect (RIBE) induction. Fraction of p53BP1 positive cells (**A**); foci number per cell (**B**); and MN frequency (**C**) in the bystander cells with or without NS 398 (50 µM) or AG (1 mM) treatment. Data are pooled from at least three independent repeats and the results are presented as mean ± SD. Significances in the differences between the samples are determined and differences with *p* < 0.05 are considered statistically significant.

Previous studies have reported that induction of RIBE was mediated by various signaling pathways. In the present investigation, we examined the influence of CO (CORM-2) on the expression of iNOS and COX-2, which have been proven to be the critical mediators in RIBE signal(s) induction [24,25]. *N*-[2-(cyclohexyloxy)-4-nitrophenyl]-methanesulfonamide (NS 398, 50 μM) (inhibitor of COX-2) or aminoguanidine (AG, 1 mM; inhibitor of iNOS) was added to treat the irradiated and bystander cells 1 h before irradiation. With the treatment of NS 398, in the 2 Gy bystander cell population, the fraction of p53BP1 positive cells was reduced from 3.89% ± 0.34% to 1.49% ± 0.31% (Figure 5A), the number of foci per cell from 0.31 ± 0.03 to 0.21 ± 0.02, (Figure 5B), and the MN frequency from1.78% ± 0.09% to 0.90% ± 0.04% (Figure 5C). Similar trends for p53BP1 formation and MN frequency were also observed in the bystander cell population with AG treatment (Figure 5A–C). However, no distinct changes in the p53BP1 formation and MN frequency were observed in the irradiated cell population upon treatment with iNOS or COX-2 inhibitors (Figure S2). 

### 2.6. CO (CORM-2) Decreased Expression of iNOS and COX-2

**Figure 6:**
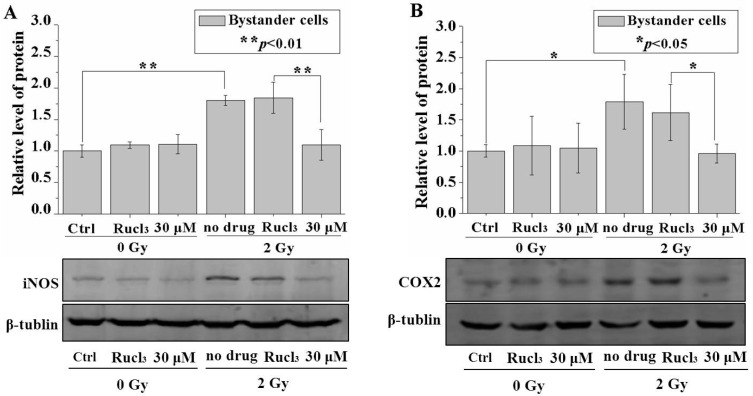
CO decreases the expression of iNOS or COX-2 in the bystander cells. Relative level of iNOS (**A**); and COX-2 (**B**) protein expression in bystander cells with or without CO (CORM-2) treatment. Data are pooled from at least three independent repeats and the results are presented as mean ± SD. Significances in the differences between the samples are determined and differences with *p* < 0.05 are considered statistically significant.

Considering that the fraction of bystander cells was 99% in the mixed cell population, the change in the overall protein level in the mixture should have been effectively attributed to the bystander cells. The results in Figure 6A,B showed that irradiation with a dose of 2 Gy increased the iNOS and COX-2 protein levels in the cell cluster, and treatment with 30 µM CO (CORM-2) significantly reduced the levels of both proteins. No distinct changes in the iNOS or COX-2 protein expression were observed in the irradiated cell population upon treatment with 30 µM CO (CORM-2) (Figure S3).

## 3. Discussion

Persaud et al. demonstrated the presence of intercellular communication between CHO cells in the cell-cluster model constructed by a cluster of mixed irradiated and bystander cells [5]. Free reactive radicals and gap junctions were found to mediate RIBE which induced mutagenesis in the bystander A_L_ cells [5]. In the present study, RIBE and the effect of CO were investigated using the same cell-cluster model. Considering that Gerashchenko’s study [23] proved 5% irradiated cells did not affect the measurement of proteins in the bystander cells, the fraction of irradiated cells in our experiment should be less than 5%. When Eric Hall’s group adopted the mixed cell cluster model to assess the mutagenicity of bystander A_L_ cells, they mixed the irradiated and bystander cells in a ratio of 1:5 to form the cluster and then separated the bystander A_L_ cells with magnetic separation twice for the further mutagenicity analysis [5]. They determined that the purity of bystander A_L_ cells was 99.24%, which was measured with flow cytometry. Furthermore, Nagasawa and Little proved that even irradiation of cell nuclei of 1% of CHO cells could efficiently induce bystander effect [26]. Taking these results altogether, the fraction of irradiated cells was set as 1%. Irradiation of 1% of the cells with a dose of 2 Gy led to a distinct increase in the amounts of DSBs (surrogated by p53BP1 foci) and chromosome aberration (surrogated by MN induction) in the other 99% bystander cells in the mixed cluster. Furthermore, the bystander cells exhibited a significant increase in the cell growth rate even when the genetic damages remained unrepaired. RIBE stimulating proliferation was first reported by Iyer et al. [8] and was also observed by other groups [27,28]. Positive effects of RIBE were also reported such as reduced neoplastic transformation [13], overexpressed protective proteins in bystander fish [14] and in rats [15], and induced adaptive response [16,17,18]. Gerashchenko et al. considered that direct contact between irradiated and unirradiated bystander cells was very important for enhancing proliferation since the low densities of irradiated and bystander cells in the mixing culture and separation between irradiated and bystander cells with transwell inserts did not induce significant proliferation in the bystander cell population [29]. In our model of cell cluster, the irradiated and bystander cells were mixed close together to ensure direct contacts. Contradictory biological effects induced by RIBE from different research models indicated that the mechanisms underlying RIBE were complicated. CDC2 is a protein kinase that complexes with mitotic cyclins and is required for mitotic entry. CDC2 might also be altered in a manner that would favor an enhanced state of proliferation [30]. We detected an increased level of CDC2 in the bystander cells and this result was commensurate with those of Iyer et al*.* [8]. Radiation-induced down-regulation of the CDC2 level through induction of p53 and p21 proteins was revealed by Azzam et al. [31]. Our results also showed decreased levels of CDC2 in the irradiated cells. 

Our previous studies revealed that low concentrations of CO could modulate or inhibit RIBE in monolayer cultured cell populations [14,15,16]. In the present work, further research was performed using a cell-cluster model. Our results showed that a low concentration (30 μM) of CO (CORM-2) could effectively reduce DSB in bystander cells, MN formation and cell growth back to the control level. On the other hand, the effects of direct irradiation (with a dose of 2 Gy) were not changed after treatment with CO (CORM-2). On comparison with previous results from monolayer cell cultures [14,15,16], the concentration of CO (CORM-2) which can effectively modulate the RIBE increased from 20 to 30 μM. This might be due to the difference in the monolayer and cluster models. Previous research on RIBE revealed higher frequencies of apoptosis [32,33] and mutagenesis [5,34] in 3-D models than those in monolayer cultured cell models. These results suggested that the high concentration of CO could be explained because the gas needed to go several layers deeper now, compared to a monolayer of cells.

Studies on mechanisms underlying RIBE revealed that activation of iNOS or COX-2 played critical role(s) in RIBE [1,35]. Upregulation of iNOS, which led to the production of reactive nitrogen species in the bystander cells, was observed as the downstream of mitogen activated protein kinase (MAPK) pathways [25] or cytokines such as TGF-β1 released by irradiated cells [36]. COX-2, a downstream factor of the important transcription factor NF-κB, was also reported to mediate the transduction of stress signaling pathways in bystander cells [37,38]. Modulation of iNOS or COX-2 by low concentrations of CO was reported previously. Cavicchi et al. proved that CO acted at the transcriptional level to inhibit iNOS expression in the human intestinal epithelial cell line DLD-1 [39]. The results of Zuckerbraun et al. also showed that CO decreased cytokine- or hypoxia/endotoxin-induced iNOS/NO production in cultured IEC-6 cells, which was a rat enterocyte cell line [40]. Chien et al. found that thrombin-induced COX-2 expression was significantly attenuated by pretreatment with CORM-2 (30 μM) [41]**.** Suh et al. reported that CO downregulated lipopolysaccharide-induced COX-2 mRNA and protein expression and iNOS expression in murine macrophage [42]. Uddin et al. reported that CO pretreatment inhibited GSK-3β activation and decreased TNF-α and iNOS expression by inhibition of NF-κB activation in LPS-stimulated U937 and Mesenteric Lymph Node cells [43]. 

In the present work, treatment with CO (CORM-2) decreased the protein expression of iNOS and COX-2 in the bystander cells but not in the irradiated cells in the multicellular cluster model. Combining the results in the present work as well as those obtained in our previous studies, it is concluded that CO might inhibit RIBE via modulating these two critical RIBE signaling mediators, which activated their downstream effect to cause DNA or chromosome damage. More studies, e.g., in vivo studies of inhibiting RIBE with low concentrations of CO in mouse models, on the mechanism underlying the modulation of RIBE by CO should be performed in the future.

Finally, a low concentration of exogenous CO has potential clinical uses in protecting against cell death, anti-inflammation, protection against oxidative injury, protection against acute lung injury, and enhancement in the tolerance of organ transplantation etc. [13]. Our present results and further studies in this direction can help mitigate RIBE-induced hazards during radiotherapy procedures.

## 4. Materials and Methods

### 4.1. Cell Culture and X-ray Irradiation

CHO cells were cultured in a mixed medium (Dulbecco’s Modified Eagle Medium:F-12 = 1:1) (Hyclone, Logan, UT, USA) supplied with 10% fetal bovine serum (FBS; Hyclone, Logan, UT, USA). The cells were maintained in a humidified atmosphere with 5% CO_2_ at 37 °C. After growing into near full confluence, the cells were irradiated (2 Gy) with an X-ray irradiator (XSZ-220/20, Kangjia, Dandong, China) at a dose rate of 1.94 Gy/min (120 kV, 12.2 mA). 

### 4.2. Preparation of Cell Cluster

The preparation of cell cluster fully followed the protocol developed by Hall’s group [5]. The RIBE, determined through DNA damage in bystander cells, was proven to be fully induced since the cluster allowed intimate contact between the cells and formation of functional gap junction even though the cell cluster was maintained at 11 °C for 24 h [5,34]. The protocol is described briefly below: The cells destined to be irradiated were stained with 5 μM CellTracker Orange CMRA (Invitrogen, Eugnene, OR, USA) in the culture medium for 30–45 min immediately before irradiation. The cells were then washed with warmed PBS twice to remove the excessive dye.

Immediately after irradiation, the irradiated and non-irradiated cells were trypsinized, and 1 × 10^4^ irradiated and 9.9 × 10^5^ non-irradiated cells were mixed in a ratio of 1:99. The cell mixture was then centrifuged (94× *g*) for 1 min to produce a cluster with 1 mL medium in a microcentrifuge tube. The cluster was then maintained at 11 °C for 24 h to allow the possible transfer of bystander signals from irradiated cells to neighboring non-irradiated bystander cells [5]. Then the cluster was resuspended and the mixed cells were plated on the cover glasses for immunofluorescence and MN tests.

### 4.3. CO (CORM-2) Treatment

CO was generated by the CO releasing molecule, [Ru(CO)_3_Cl_2_]_2_ (CORM-2, Sigma-Aldrich, St. Louis, MO, USA), which released CO when dissolved in the medium [44]. The stock solution (50 mM) was freshly prepared by dissolving CORM-2 in dimethyl sulfoxide (DMSO, Sigma-Aldrich, St. Louis, MO, USA). For each mole of CORM-2, 0.7 mole of CO was liberated [44]. Control experiments were performed by using RuCl_3_ instead of CORM-2 dissolved in DMSO. The cell populations (both irradiated and non-irradiated bystander populations) were treated with or without CORM-2 for 1 h before irradiation and the chemical would be present in the culture system until the end of multicellular cluster culture. 

### 4.4. Immunofluorescence of p53 Binding Protein 1 (p53BP1) 

We employed fluorescence detection of foci formation ofp53BP1 as a marker of DSB [45]. After incubation of irradiated and bystander CHO cells in the cluster for 24 h, the mixed cells were washed with PBS twice and 5 × 10^4^ cells were plated on cover glasses. At 5 h after cell plating, the cells were fixed with 2% buffered paraformaldehyde for 20 min at room temperature, and were then rinsed three times with PBS again. Prior to immunochemical staining, the cells were incubated for 20 min in TNBS solution (PBS supplemented with 0.1% Triton X-100 and 1% FBS) to improve their permeability. The cells were then incubated with rabbit anti-p53BP1 antibody (Abcam, Cambridge, MA, USA) at 1:200 in PBS+ (PBS supplemented with 1% FBS) for 60 min, washed with TNBS for 3 × 5 min, and then incubated in PBS^+^ containing secondary goat anti-rabbit IgG-FITC (Santa Cruz, CA, USA) for a further 60 min. After another wash with TNBS for 3 × 5 min, the cells were counterstained with Hoechst 33342 at a concentration of 20 µg/mL for 20 min at room temperature. After a final wash with TNBS, at least 400 cells were counted using a fluorescent microscope (Leica DMI 4000B, Wetzlar, Germany). The cells containing two or more p53BP1 foci were regarded as positive cells.

### 4.5. MN Assay

After incubation of the cells in the cluster for 24 h, the cells were washed twice with PBS and then sub-cultured in 35 mm Petri dishes (Nunc, Kamstrupvej, Denmark). The medium was replaced with fresh medium containing 1.5 μg/mL cytochalasin-B (Sigma-Aldrich, St. Louis, MO, USA) at 5 h after cell seeding, and the cells were cultured for a further 24 h. The cells were then fixed with 2% paraformaldehyde (Sinopharm Chmemical Reagent, Shanghai, China), and stained with DAPI (Beyotime, Shanghai, China). The MN in bi-nucleated (BN) cells were morphologically identified and more than 1000 BN cells were scored for each sample.

### 4.6. Cell Proliferation Assay

Cell proliferation was assayed by counting the cell numbers. After incubation of irradiated and bystander CHO cells in the cluster for 24 h, the mixed cells were washed with PBS twice and 3 × 10^5^ cells were seeded in 35 mm Petri dishes. The cell number was then counted with Cell Counter (Count star) at 24 h and 48 h after cell seeding.

### 4.7. Western Blot Analyses

After incubation of cell cluster for 24 h, the cells were washed twice with PBS and were then sub-cultured in 35 mm Petri dishes for 24 h. Total protein was extracted. SDS-polyacrylamide gel electrophoresis and Western blotting were then performed. Samples (40 µg of protein) were subjected to 10% SDS-PAGE, transferred onto PVDF membranes and assayed for CDC2 (p34^cdc2^) (Santa Cruz, CA, USA), cyclooxygenase-2 (COX-2), inducible nitric oxide synthase (iNOS) and β-tublin (Cell Signaling, Boston, MA, USA) protein expression using the ECL kit (Kangweishiji Biotechnology, Beijing, China) according to the manufacturers’ instructions. The relative levels of CDC2, COX-2 or iNOS proteins were measured with densitometry and analyzed with the software Image J.

### 4.8. Statistical Analysis

Statistical analysis was performed on data obtained from at least three independent experiments. All results were presented as means ± SD. The significance level was assessed using ANOVA Tukey *post-hoc* test and *p* values smaller than 0.05 were considered statistically significant.

## Supplementary Materials

Supplementary materials can be found at www.mdpi.com/1422-0067/17/12/2051/s1.

## Abbreviations

RIBE	radiation-induced bystander effect
CO	carbon monoxide
CORM-2	CO releasing molecule, tricarbonyldichlororuthenium
DSB	DNA double-strand breaks
MN	micronucleus
iNOS	inducible nitric oxide synthase
COX-2	cyclooxygenase-2
DMSO	dimethyl sulfoxide
p53BP1	p53 binding protein 1
RuCl_3_	ruthenium trichloride
CDC2	cell division cycle 2
NS 398	*N*-[2-(cyclohexyloxy)-4-nitrophenyl]-methanesulfonamide
AG	aminoguanidine
MAPK	mitogen activated protein kinase
