# Supplementary material for: Low Concentration of Exogenous Carbon Monoxide Modulates Radiation-Induced Bystander Effect in Mammalian Cell Cluster Model
Source: Int J Mol Sci. 2016 Dec 8;17(12):2051. doi: 10.3390/ijms17122051 (PMC5187851; doi:10.3390/ijms17122051)
Supplement: Supplementary file 1 [file ijms-17-02051-s001.pdf]

Supplementary Materials: Low Concentration of Exogenous Carbon Monoxide Modulates Radiation-Induced Bystander Effect in Mammalian Cell Cluster Model

Wenqing Wu, Lili Nie, K. N. Yu, Lijun Wu, Peizhong Kong, Lingzhi Bao, Guodong Chen, Haoran Yang and Wei Han

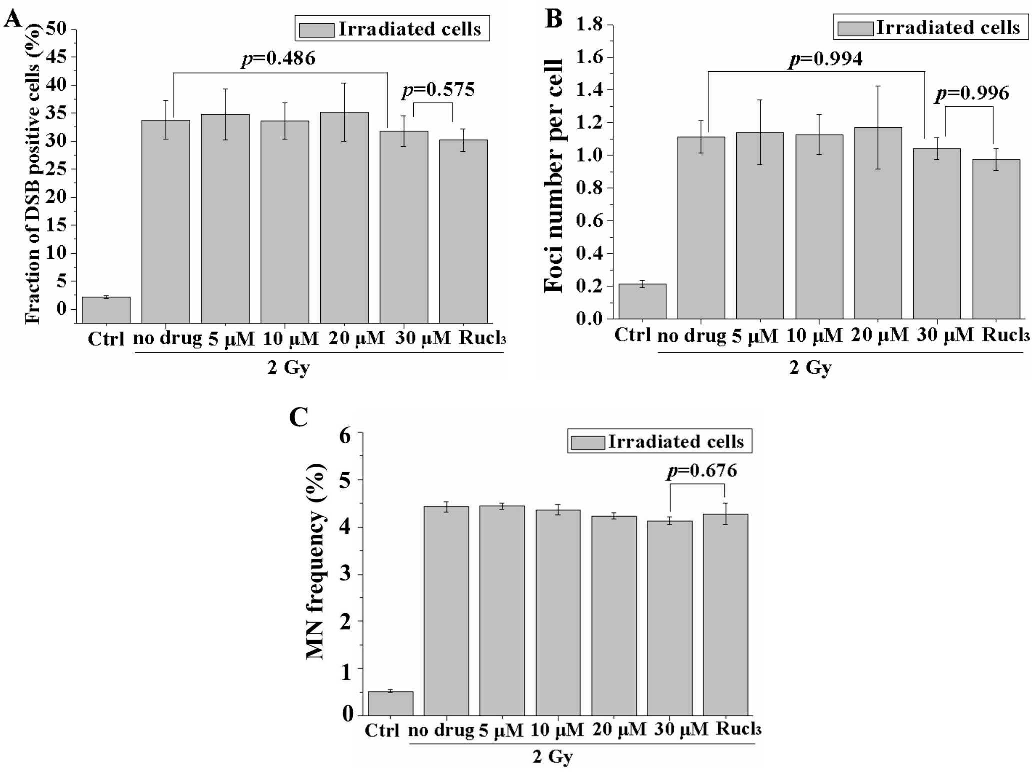

Figure S1. CO did not affect DSB formation in the irradiated cells. Fraction of p53BP1 positive cells (**A**); foci number per cell (**B**); and MN frequency (**C**) in the irradiated cells with or without CO (CORM-2) treatment. Data are pooled from at least 3 independent repeats and the results are presented as mean ± SD. Significances in the differences between the samples are determined and differences with $p < 0.05$ are considered statistically significant.

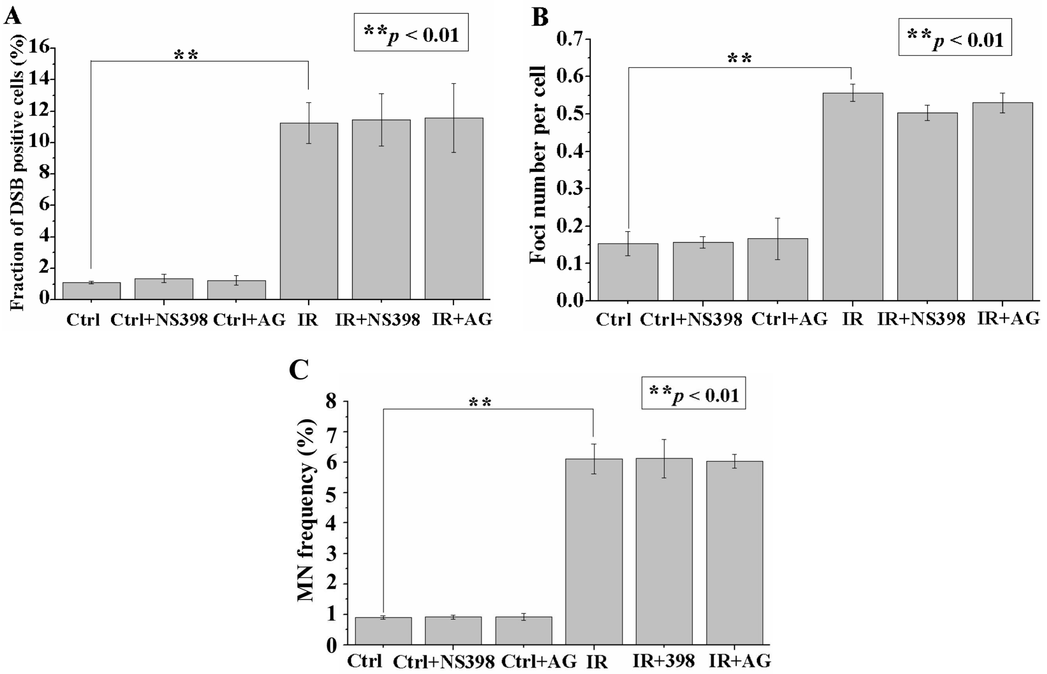

Figure S2. NS 398 or AG did not affect DSB and MN formation in the irradiated cells. Fraction of p53BP1 positive cells (**A**); foci number per cell (**B**); and MN frequency (**C**) in the irradiated cells with or without NS 398 (50 µM) or AG (1 mM) treatment. Data are pooled from at least 3 independent repeats and the results are presented as mean ± SD. Significances in the differences between the samples are determined and differences with $p < 0.05$ are considered statistically significant.

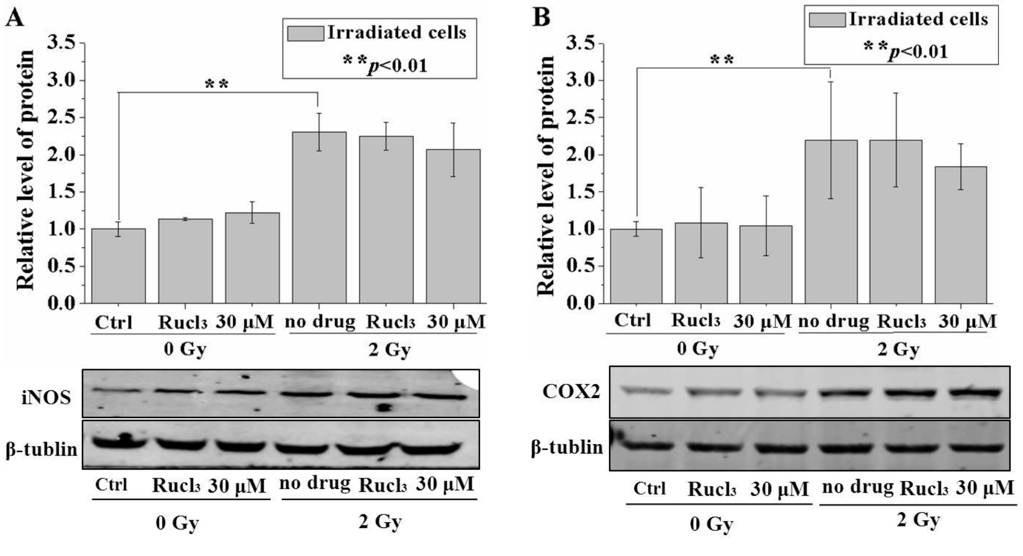

Figure S3. CO did not affect the expression of iNOS or COX-2 in the irradiated cells. Relative level of iNOS (**A**); and COX-2 (**B**) protein expression in irradiated cells with or without CO (CORM-2) treatment. Data are pooled from at least 3 independent repeats and the results are presented as mean ± SD. Significances in the differences between the samples are determined and differences with $p < 0.05$ are considered statistically significant.
